# Targeting ncRNAs in the 3q26.2 amplicon

**DOI:** 10.18632/oncoscience.221

**Published:** 2015-08-31

**Authors:** Pradeep Chaluvally-Raghavan, Gordon B. Mills

**Affiliations:** Department of Systems Biology, The University of Texas MD Anderson Cancer Center, Houston, TX, USA

**Keywords:** CNA, microRNA, miR-therapy, TP53INP1, ovarian cancer

Whole human genome sequencing studies have revealed that the protein coding portion of the genome accounts for only about 2% of the genome [1]; this warrants the exploration of the role of the larger noncoding portion of the genome, which was previously considered “junk DNA”. Noncoding RNAs (ncRNAs), including microRNAs, have been relatively unexplored as potential tumor drivers since they were first identified over a decade ago [2]. Advances in ncRNA research suggest that miRNAs are not only drivers of oncogenesis but represent emerging therapeutic targets to inhibit tumor growth and metastasis [3]. Our recent studies demonstrated that the 3q26.2 amplicon leads to increased expression of an oncogenic microRNA, miR569 [4]. In this brief report, we summarize the role of the 3q26 amplicon in the regulation of oncogenic microRNA miR569. We also provide evidence for novel therapeutic opportunities and improved chemotherapy sensitivity by inhibiting miR569 activity.

Studies from our laboratory and others have revealed that copy number aberrations (CNA) of 3q26.2 lead to aberrant expression of a set of potential oncogenes including PIK3CA, PIK3CB, PKC, SNON, MECOM as well as TERC in ovarian and other cancers [5], suggesting that multiple components in the amplicon contribute to tumor initiation and progression either alone or in cooperation. Recently, we found that miR569, which is regulated by the 3q26.2 amplicon, deregulates a critical viability pathway by inhibiting the expression of tumor protein p53-induced nuclear protein 1 (TP53INP1), which in turn increases the proliferation and survival of ovarian cancer cells. TP53INP1 transcription is primarily regulated by the p53 protein; however, other transcription factors such as p73 or NF-κB can regulate the expression of TP53INP1 during cellular stress [4]. However, the long 3′UTR of TP53INP1 human mRNA (~4500 base pairs) conveys the potential for miRNA-mediated regulation of TP53INP1 mRNA.

TP53INP1 binds to homeodomain-interacting protein kinase 2 (HIPK2), which phosphorylates p53 on Ser-46 [6]. Phosphorylation of p53 triggers the transcription of several downstream effectors such as P53AIP1, MDM2, p21, PIG3, and BAX, thereby leading to G1 cell cycle arrest or apoptotic cell death [7]. Indeed, loss of TP53INP1 has been associated with coordinate decreases in several tumor suppressors including P53AIP1, caspase-3, and p21, and impaired cell cycle regulation and apoptosis [8]. The decrease in TP53INP1 expression during preneoplastic development as well as in many types of cancers may interrupt the function of p53 [4, 9]. Furthermore reduced TP53INP1 correlates with tumor progression and poor outcomes, thereby suggesting that TP53INP1 mRNA or protein levels might be an indicator of disease outcome in cancer patients.

Based on our results, the miR569 onco-miR is expressed at high levels in ovarian cancer patient's samples and cell lines enabling cellular transformation, proliferation, and metastasis. Therefore, microRNA inhibitors have the potential to act as novel therapeutic agents. To inhibit miR569, we used single-stranded RNA molecules called anti-miRs designed to specifically bind to the endogenous miR569 molecules to downregulate miR569 expression and activity. As expected anti-miR569, rescued TP53INP1 expression and further augmented p53 phosphorylation on Ser46 and expression of P53AIP1, Bax, and p21, which in turn induced apoptosis of ovarian cancer cells *in vitro*. Importantly, knockdown of TP53INP1 completely reversed cell death and caspase-3 activity induced by anti-miR569, consistent with the contention that TP53INP1 is the key target of miR569. Overexpression of miR569 was able to partially reverse the effects of anti-miR569, suggesting the specificity of the effects of anti-miR on its target miR569.

Advances in our understanding of microRNAs as critical regulators in a wide variety of diseases including cancer in recent years, have provided new opportunities to target miRNAs using antimiRs as an effective mechanism to restrain the effects of genes and pathways in cancer. Indeed, RNA interference (RNAi)-mediated gene silencing is rapidly emerging as a therapeutic approach. Early trials are underway with several demonstrating safety or clinical responses in cancer patients with RNAi therapies targeting mRNAs that encode protein kinase N3 (PKN3) Polo kinase 1 (PLK1), vascular endothelial growth factor (VEGF) and kinesin spindle protein (KSP), the K-Ras G12D mutant, and the M2 subunit of ribonucleotide reductase (RRM2) [3].

To test the effects of anti-miR569 *in vivo,* we incorporated anti-miR569 into neutral liposomes (1,2-dioleoyl-sn-glycero-3-phosphotidylcholine-DOPC). Remarkably, anti-miR569 reduced tumor weight and number of tumor sites in the peritoneal cavity of tumors that overexpressed miR569 but not tumors with low levels of miR569. Strikingly, the combination of anti-miR569 with cisplatin, the standard first line treatment for ovarian cancer, resulted in a massive decrease in tumor weight and the number of tumor sites in the peritoneal cavity. Therefore, treatment with target-specific anti-miRs either alone or in combination with cytotoxic drugs or small molecule inhibitors has the potential to improve the efficacy of cancer treatment in preclinical and clinical studies.

**Figure 1:**
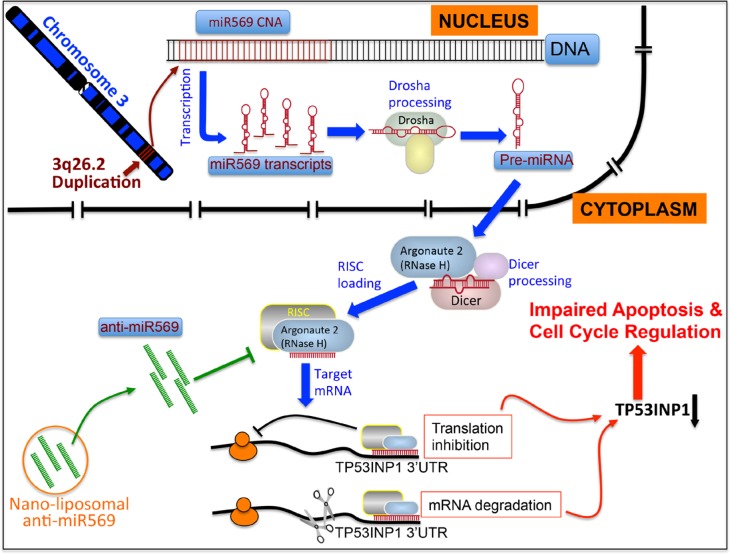
Model demonstrates that 3q26.2 CNA leads to increased miR569, downregulates TP53INP1, causes impaired apoptosis and cell cycle regulation

In summary, recent research coupled with our studies suggest that the regulation of miRNAs provides an exciting emerging opportunity to treat various diseases, including cancer. Target specific anti-miRs and novel chemistries to incorporate into nanovectors with or without selective targeting of nanovectors to the tumor are under way to further evaluate these entities as suitable drug candidates for preclinical and clinical studies.
